# Nanomaterial based aptasensors for clinical and environmental diagnostic applications

**DOI:** 10.1039/c9na00153k

**Published:** 2019-04-29

**Authors:** Harmanjit Kaur, Munish Shorie

**Affiliations:** Institute of Nano Science and Technology Mohali 160062 India munish_shorie@yahoo.com

## Abstract

Nanomaterials have been exploited extensively to fabricate various biosensors for clinical diagnostics and food & environmental monitoring. These materials in conjugation with highly specific aptamers (next-gen antibody mimics) have enhanced the selectivity, sensitivity and rapidness of the developed aptasensors for numerous targets ranging from small molecules such as heavy metal ions to complex matrices containing large entities like cells. In this review, we highlight the recent advancements in nanomaterial based aptasensors from the past five years also including the basics of conventionally used detection methodologies that paved the way for futuristic sensing techniques. The aptasensors have been categorised based upon these detection techniques and their modifications *viz.*, colorimetric, fluorometric, Raman spectroscopy, electro-chemiluminescence, voltammetric, impedimetric and mechanical force-based sensing of a multitude of targets are discussed in detail. The bio-interaction of these numerous nanomaterials with the aptameric component and that of the complete aptasensor with the target have been studied in great depth. This review thus acts as a compendium for nanomaterial based aptasensors and their applications in the field of clinical and environmental diagnosis.

## Abstract 2

Exploring nanomaterials for the fabrication of aptamer based biosensors and their application in clinical diagnostics and contaminant monitoring in food & environment.

## Graphical abstract

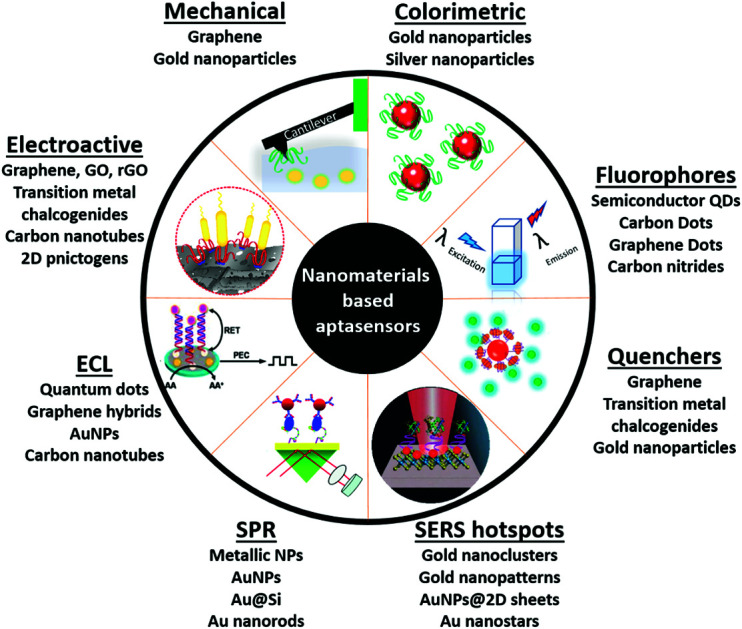

## Introduction

The history of sensors dates back to 1909 with the development of a pH sensor by Søren Sørensen.^1,2^ However, true ‘biosensors’ were introduced in 1962 by Leland C. Clark, whose work laid the foundation for the invention of glucose sensing platforms.^3,4^ A biosensor in a conventional notion comprises a biological component (*viz.*, cells, enzymes, antibodies, peptides, oligonucleotides, *etc.*), whose interaction with the target analyte yields a physical or chemical change that is amplified into a readable signal with the help of a transducer component.^5–8^ As efficient bio-receptors, enzymes have a limited repertoire and were replaced by antibodies, which conferred target flexibility giving a forward thrust to the biosensing industry. Conventional enzyme-based biosensors have the advantage of signal amplification mainly relying on the substrate concentration and enzyme turnover number. This missing inherent property was amended by adding enzymatic label molecules in antibody-based diagnostics that transduces the bio-molecular interaction into a readable signal. The excellent selectivity of antibodies has found compromise in the current market with a low shelf life and temperature sensitivity.^9–11^ In 1990, Tuerk & Gold and Ellington & Szostak independently published seminal papers discussing the possibilities of selecting nucleic acid-based receptors *in vitro* from large random libraries.^12,13^ These receptors called aptamers (a term with Latin and Greek origins: *aptus* meaning fit and *meros* meaning part) have shown notable moldable selectivity for diverse target analytes, ranging from small inorganic molecules, ions, sugars, proteins, and viruses to cells.^14–33^ Such a varied target range is lacking in antibody generation due to the restriction of target receptor exposure in their native forms. This is more evident in whole cells where the surface antigens contain certain hidden trans-membrane regions, a fact which is generally not accounted for during the maturation of antibodies. In contrast, aptamers are generated against targets in their naturally occurring states due to their *in vitro* generation route.^34,35^ Over the past three decades they have successfully cemented their superiority over antibodies due to several factors, *viz.*, *in vitro* generation, ease of maintenance & amplification, ability to be generated against small or non-protein molecules, and above all the compatibility of the process with toxic molecules.^10,34,36–39^ This *in vitro* generation process called **S**ystemic **E**volution of **L**igands by **EX**ponential enrichment (SELEX) is an iterative method, which relies on the repetitive exposure of naïve oligomer molecules to target populations followed by screening and re-exposure of binding oligomers creating a selection pressure to enrich high affinity ligands to the target analyte. These high affinity screened oligomers are then aptly labelled “Aptamer” for that particular target. Over the years a multitude of SELEX variants have been developed, *viz.*, cell-SELEX,^35^ capillary electrophoresis based SELEX,^40,41^ microfluidics-SELEX,^42,43^ FACS based SELEX,^44^ microtitre plate-SELEX,^45,46^ magnetic bead SELEX,^47^ and *in vivo* SELEX.^48,49^ Each method has been developed considering the final application of the aptamer with respect to the target of interest and various researchers have successfully adopted several modifications.^50^

**Fig. 1:**
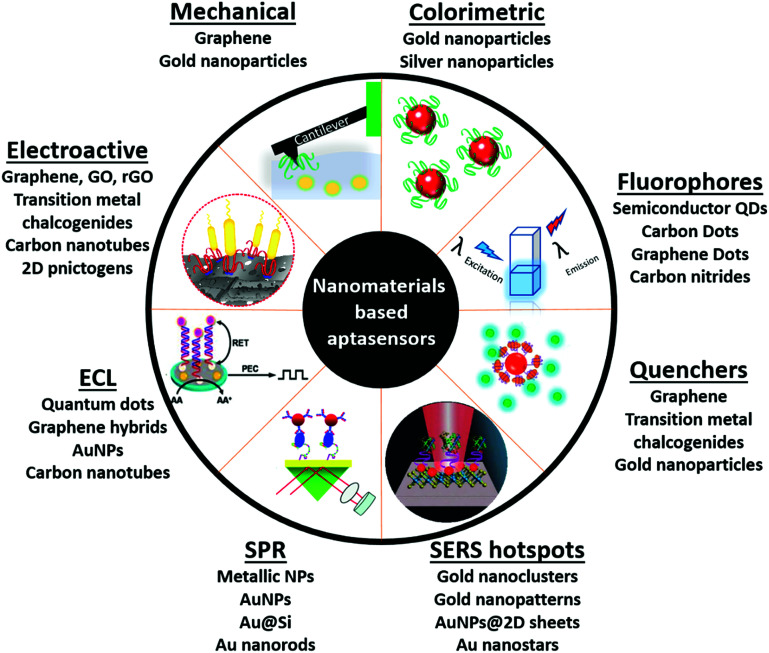
Schematic representation of nanomaterials' applications as transducers for the fabrication of aptasensors.

With the emergence of nanotechnological tools, numerous materials with enhanced optical, electrical, mechanical and electrochemical sensitivities have been successfully synthesized.^51^ Such nanomaterials have been used directly in several label-free formats as they possess the ability to generate a high signal to noise ratio even at very low substrate concentrations. Antibodies, due to their large sizes generally lead to (i) the formation of large insulating layers in the case of electrochemical sensors, (ii) large mass increments in the case of mechanical sensors, and (iii) a great increase in distances between fluorophores in fluorescence based sensors, thus compromising the signals significantly.^36,38^ The application of aptamers in place of antibodies as biological recognition elements in these sensitive nanotransducers enhances their functioning owing to the smaller size of aptamers. The amalgamation of aptamers and advanced nanomaterials forges platforms called aptasensors, which possess very high selectivity and superior sensitivities *versus* conventional enzyme/antibody based diagnostic platforms (Fig. 1). In this review we will focus on various aptamer modified nanosensors and their application in clinically and environmentally significant biomarkers.

## Nanomaterial based aptasensors

### Optical aptasensors

Optical biosensors have seen most prominent improvements in recent years with the involvement of aptamers and nanomaterials. Materials with remarkable optical properties have provided avenues towards ultrasensitive detection of molecules with practical applications in security, food safety, biomedical diagnosis and medicine. During the last few years, several transducing approaches like colorimetry, fluorescence, surface-enhanced Raman spectroscopy (SERS) and surface plasmon resonance (SPR) spectroscopy have been combined with aptamers for the design and development of biosensors with label-free and real-time detection of molecules.

### Colorimetric aptasensors

**Fig. 2:**
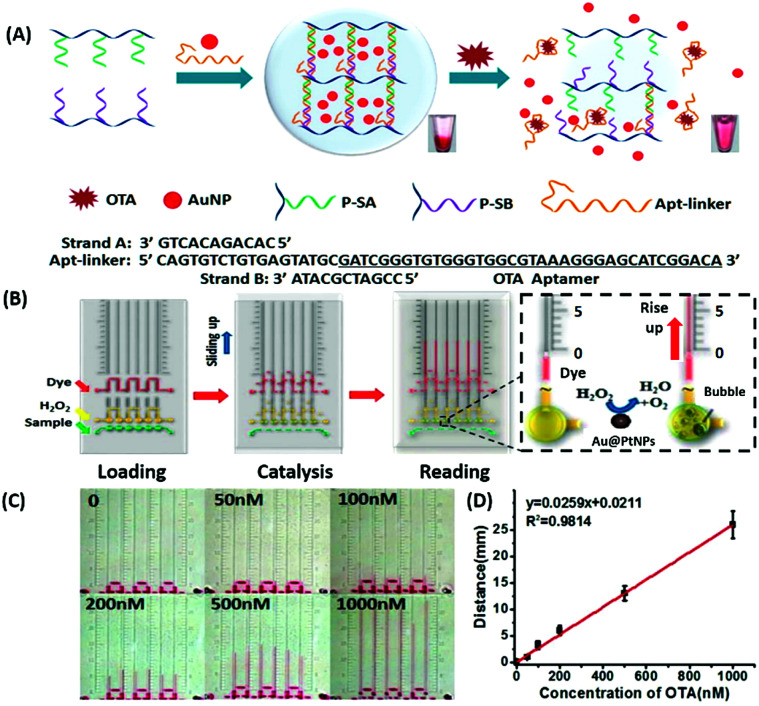
(A) Schematic representation of a gold nanoparticle encapsulated polymer–DNA hydrogel for visual detection of Ochratoxin A (OTA). The inset images show the conversion from gel to sol upon OTA addition. (B) Visual quantitative detection of OTA on a target-responsive hydrogel-based microfluidic bar-chart chip (V-chip) using platinum coated AuNPs having high catalase efficiency. The level of the ink bar is pushed by O_2_ evolved during catalysis and is proportional to the concentration of the target. (C) Images showing visual changes in ink advancement in response to OTA with a detection limit of 1.27 nM. (D) Linear standard curve for the detection of OTA. Adapted with permission from ref. 54. Copyright (2015) American Chemical Society.

**Fig. 3:**
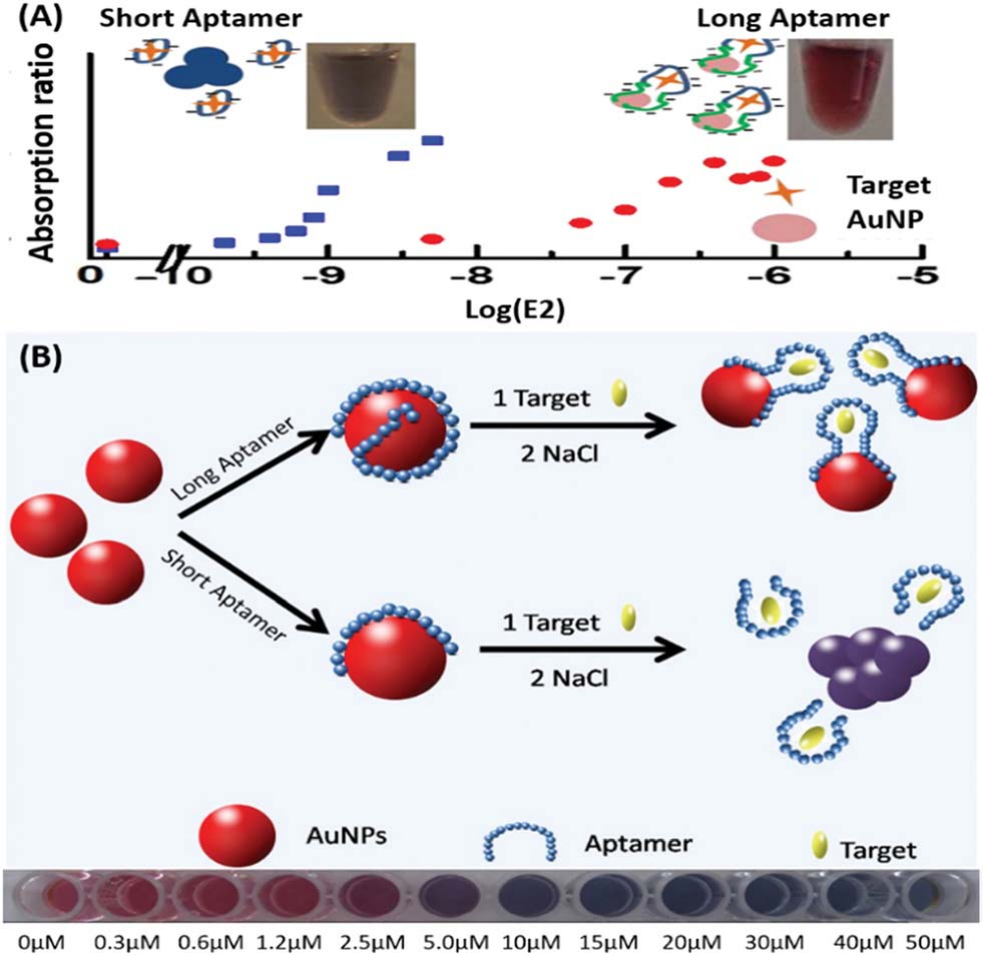
(A) Schematic representation of AuNP aggregation based 17β-estradiol aptasensing showing the effect of aptamer length on its detection range. Short aptamer strands covering AuNPs show a rapid blue shift whereas longer aptamers are unable to aggregate due to surface passivation. Adapted with permission from ref. 59. Copyright (2015) American Chemical Society. (B) Schematic representation of aptamer-wrapped AuNP based assay for acetamiprid using short aptamers optimised by the truncation method. A visible red to blue shift upon target addition from 0 to 50 μM is also shown. Adapted with permission from ref. 60. Copyright (2016) Elsevier.

Amongst optical aptasensors, colorimetric aptasensors are the most common and widely reported biosensors due to their ease of fabrication and cost-effective detection modules. In addition to the basic “signal-on” and “signal-off” types of transducers, colorimetric sensors also possess the ability of “signal-change” owing to multiple measurement points arising from a wider spectrum. This allows the designer to incorporate the measurement of signal switch from one parameter (colour) to another for the development of sensors. Gold nanoparticles (AuNPs) have been established as a standard label substance due to their simpler synthesis method, which generates nanoparticles with low poly dispersity and homogenous properties with almost no batch variations.^52,53^ Their surface plasmon gives a bright red coloration, which is highly susceptible to changes in nanoparticle size, allowing their utilization as optical probes. As transducers, AuNPs are used as both “signal-on” and “signal-change” type sensors. In “signal-on” sensors, gold nanoparticles are attached on the receptors and increase in red colour intensity is quantified as the measure of their attachment to the target analyte.^54–56^ The work of Liu & co-workers shows an interesting aptamer cross-linked hydrogel for the detection of Ochratoxin A (OTA).^54^ The platform has AuNPs trapped in a polyacrylamide–DNA hydrogel comprising two polyacrylamide–DNA conjugate units P-SA & P-SB, cross-linked *via* an OTA-specific aptamer (Fig. 2). Upon the introduction of OTA into the system, the crosslinking aptamer is dislodged from the system, releasing AuNPs during the gel–sol conversion. The system is able to detect OTA up to 1.27 nM in a microfluidic setup. The “Signal-change” type colorimetric sensors are unique and provide a wider range of applications. Gold nanoparticles of *ca.* 20 nm diameter are red in colour and show a blue shift with increase in size. The assays are designed with the aptamer providing a protective layer on the particles thus preventing salt-induced aggregation.^57,58^ Alsager *et al.* showed the use of gold nanoparticles in the detection of 17β-estradiol and highlighted the effect of DNA length on the assay functioning.^59^ They varied the aptamer length, from 75-mer, 35-mer to 22-mer, and found that truncation yielded better results until a threshold was reached beyond which the shortening resulted in a decrease of its activity (Fig. 3A). In their findings, the shorter fragments showed 25-fold improvement compared to the 75-mer parent in the detection of the analyte.^59^ In a similar study, Tian *et al.* used the truncation method to optimize the length of an acetamiprid-specific aptamer from a parent 49-mer sequence.^60^ They found that decreasing the length improved the sensitivity of the AuNP aggregation based optical assay by 3.3 fold for a 25-mer truncated aptamer (Fig. 3B). However they did not find any changes in the cross-reactivity of the aptamer suggesting the crucial role of non-binding regions in the assay designing. Another unique property of gold nanoparticles applied in colorimetric assays is their peroxidase like catalytic activity which was efficiently exploited by Weerathunge & co-workers for the detection of acetamiprid pesticide where they showed the restoration of the peroxidase-like nanozyme activity of AuNPs.^61^ The aptamer engulfed AuNPs in the presence of acetamiprid regain their catalytic activity following the displacement of aptamers from the surface of the nanoparticles. They were able to achieve a limit of detection (LoD) of 0.1 ppm in a 10 minute assay. Silver nanoparticles (AgNPs) are second to AuNPs in their applicability in colorimetric assays, which are usually based on the turning-off or turning-on of the signal.^62–64^ AgNPs lack the colour switching property and consequently the ability of usage in signal-switch type sensors. Other metal-based nanoparticles like copper nanoparticles (CuNPs), iron nanoparticles (FeNPs), and platinum nanoparticles (PtNPs) are less exploited in colorimetric assays as transducers but rather explored for other properties like catalysis or magnetism.^65–72^

### Fluorescence based aptasensors

Akin to colorimetric sensors, fluorescent platforms rely on the changes in the fluorescence signal between on and off states and in shift in wavelengths with spectrally overlapping fluorescent pairs. This adds stringency to the assay by reducing the background signal and increasing sensitivity by applying a narrow excitation source. Although the conventional readouts for fluorescence measurements are comparatively costlier, the quantifications have been easier in recent years by coupling the assay with a smartphone acting as the CCD and processing unit. This along with the usage of quantum dots (QDs) as fluorophores has brought new life to the field applicability of fluorescent sensors.

**Fig. 4:**
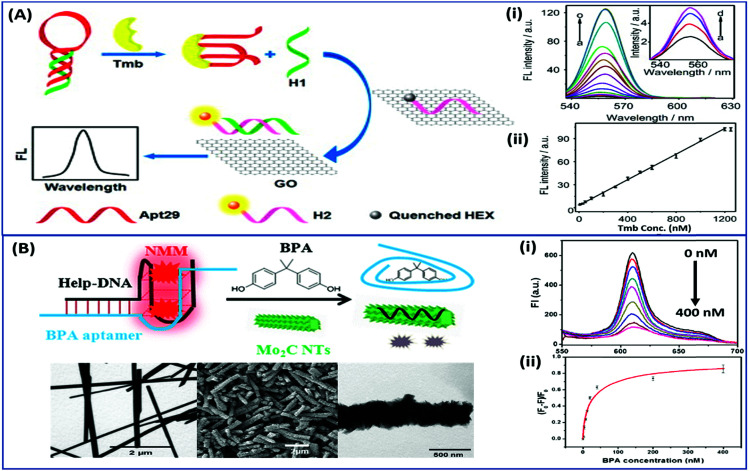
(A) Schematic representation of a “signal-on” type triple-helix aptamer as a sensing platform for thrombin (Tmb) detection using the strand-displacement method and GO nanosheets as the quencher of hexachlorofluorescein (HEX). Fluorescence spectra showing the recovery of fluorescence of the detection platform in the presence of thrombin ranging from 0 to 1250 nM (a–o). Inset shows amplified fluorescence spectra in the presence of 0–30 nM thrombin (a–d). Adapted with permission from ref. 74. Copyright (2015) Elsevier. (B) Schematic representation of a “signal-off” type aptasensing platform for the detection of BPA using Mo_2_C nanotubes. Electron micrographs showing the morphology of the surface of nanotubes before and after BPA exposure. The fluorescence spectra showing a decrease in the signal in the presence of 0–400 nM BPA (i) and its calibration curve (ii). Reprinted with permission from ref. 81. Copyright (2017) Springer Nature.

**Fig. 5:**
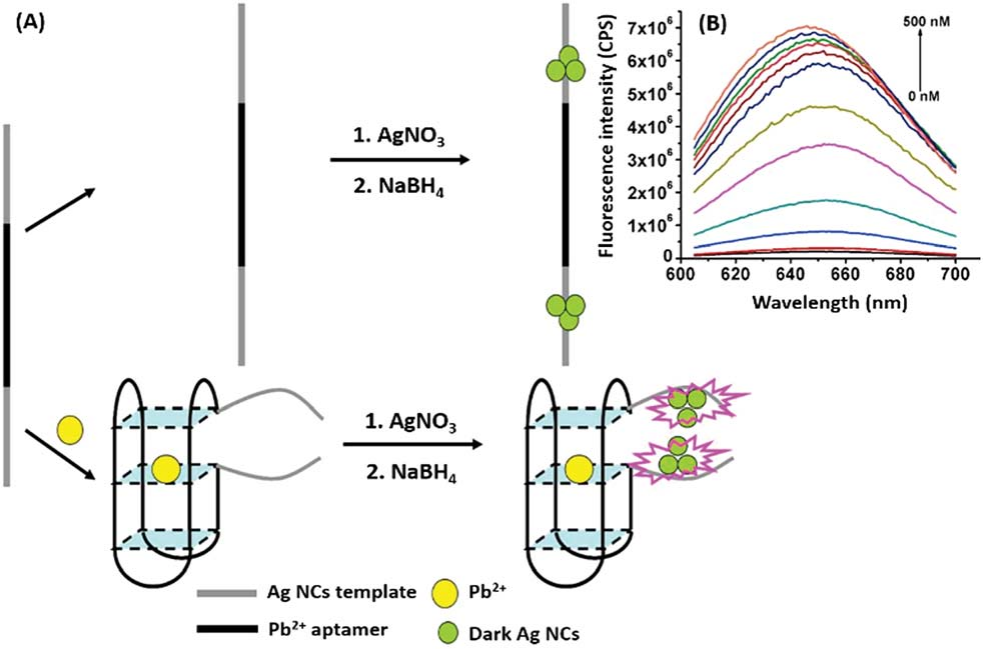
(A) Schematic representation of apta-assay for lead ions using ssDNA scaffolded fluorescent silver nanoclusters by utilising target-induced conformation change in aptamers. (B) Fluorescence spectra showing changes in AgNC fluorescence in the presence of lead ions in a concentration range of 0–500 nM. Adapted with permission from ref. 92. Copyright (2018) Elsevier.

Nanomaterials may be applied in two distinct ways in the designing of such aptasensors by either acting as fluorophores or quenchers. A nanomaterial as a quencher is the more commonly followed route wherein mainly graphene oxide (GO) has been utilized due to its ease of functionalization for conjugation, non-toxic nature, and high surface area which ultimately creates the most superior candidate for such applications. The planar structure of GO provides a very large surface area for molecular interactions and is able to harvest the radiative energy emitted by the fluorophores, quenching them in the process. Several researchers follow this approach for strand-displacement assays by applying the natural tendency of single-stranded aptamers to bind with planar sheets. It has been found that DNA in its single stranded form is quite flexible and is able to interact with planar sheets freely. However, binding to analytes creates target-induced conformational changes in aptamers, increasing their rigidity and preventing them from binding to nanosheets. This fact is employed in the development of strand-displacement based apta-assays using 2D nanomaterials, commonly graphene oxide, where the fluorophore–aptamer conjugate remains bound to GO in the quenched state in the absence of target moieties. Addition of target molecules to the system detaches the strands from GO restoring the fluorescence leading to an increase in the signal. While the work of Zhu & co-workers applied a more conventional approach of using graphene oxide (GO) as a quencher in a green emissive fluorophore modified aptamer,^73^ the work of Xu *et al.* & Ling *et al.* revolved around a more novel “turning-on” signal, in an intricate play between the aptamer and its complementary fluorophore-labelled helper DNA.^74,75^ While their interaction in control samples keeps the helper DNA from interacting with nanosheets, the introduction of target molecules displaces the helper DNA from aptamers leading to their absorption on the surface of nanosheets causing it to quench (Fig. 4A). The paradigm shift following the developments in the generation of 2D nanomaterials by facile methods like physical exfoliation created a class of planar nanostructures apart from the graphene family.^76,77^ In their work, Kong & colleagues compared the ability of GO and MoS_2_ in the detection of PSA, a prostate cancer biomarker, and found the superiority of MoS_2_ in their experiments.^78^ Apart from GO, gold nanoparticles are found to be excellent quenchers of radiative energy due to their surface plasmons, as shown in the studies of Jiang *et al.* & Ling *et al.* They studied the cleavage of the binding pocket of a RNA aptamer in two fragments where one fragment was modified with a fluorophore while the other was conjugated to AuNPs. The exposure to their respective targets crosslinks the two strands together inducing a quenching of the fluorophore due to energy transfer to AuNPs.^79,80^ In another study, use of molybdenum carbide nanotubes (Mo_2_C NTs) is shown by He & co-workers for the detection of bisphenol A (BPA).^81^*N*-Methylmesoporphyrin IX (NMM), a fluorophore stacked in the aptamer, is displaced in the presence of BPA and is physically absorbed by Mo_2_C NTs leading to a decrease in the fluorescence signal (Fig. 4B). With more nanomaterials being added to the family of 2D materials, it would not be surprising to find more similar studies to find the best-suited candidate in such bioassays. Other prominent nanomaterials used in fluorescence assays are 0D family nanomaterials called quantum dots (QDs). Quantum dots are nanoparticles of diameters smaller than 5 nm, possessing brilliant luminescence properties as a virtue of the quantum effects arising due to their tiny sizes. The fluorescence of QDs can usually be controlled by their size^82,83^ and shape,^84^ or by carefully tweaking the precursors and reaction conditions.^85–88^ Due to their high luminosity, QDs are utilized as fluorescent probes in conventional fluorescence assays and the signal can be measured even at ultra-low analyte concentrations owing to high signal intensity per unit analyte interaction.^89–91^ Apart from QDs, a novel “turn-on” biosensor was developed by Zhang & Wei for the detection of lead ions using silver nanoclusters (AgNCs).^92^ This highly selective, simple and sensitive assay uses a delicate construct using a lead(ii) ion binding central region flanked by silver binding regions on both ends (Fig. 5). The whole molecule folds in a G-quadruplex in the presence of Pb^2+^ ions, bringing the silver binding regions closer forming fluorescent AgNCs. The measured fluorescence increase is linear in the range of 5–50 nM Pb^2+^ ions with a LoD of 3 nM, and the aptasensing platform has low cross reactivity with other ionic species.

**Fig. 6:**
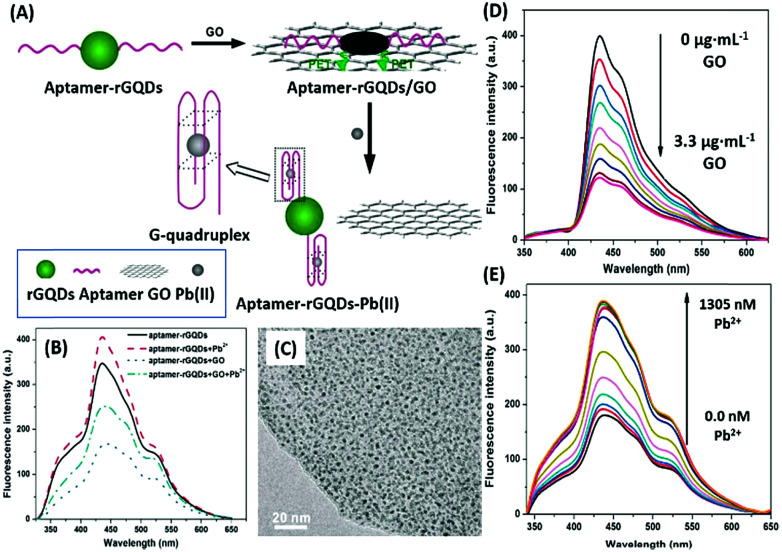
(A) Schematic illustration of the lead(ii) detection strategy of a nanosensor based on photoinduced electron transfer between graphene quantum dots and graphene oxide. (B) The quenching effect of GO nanosheets on aptamer–GQDs, and its restoration in the presence of lead ions. (C) Transmission electron micrograph of the GQD–apta–GO complex. (D) Fluorescence spectra showing quenching of GQDs in the presence of GO in a concentration range of 0–3.3 μg mL^−1^. (E) Fluorescence spectra showing restoration of fluorescence upon addition of 0–1305 nM lead ions. Adapted with permission from ref. 93. Copyright (2015) Elsevier.

Additionally, QDs can combine with another nanomaterial (usually planar) as a fluorophore–quencher pair to develop unique signal-on type assays. In a study discussing the detection of lead ions, Qian *et al.* used graphene QDs (GQDs) as the fluorescent label, which are quenched when bound to GO nanosheets (Fig. 6). The aptamer bound GQDs are displaced following the formation of a G-quadruplex in the presence of lead ions, thus restoring fluorescence up to a concentration of 400 nM.^93^ In a similar study, Wang *et al.* conjugated an aflatoxin B1 specific aptamer with AuNPs which is able to house free nitrogen-doped carbon dots, releasing them only in the presence of target molecules. The increment is used as the measure for the quantification of aflatoxin B1 in a concentration range of 16 pM to 6.4 nM having a LoD of 16 pM.^94^ Alternatively, Sabet *et al.* used AuNPs as quenchers of the fluorescence of cadmium telluride (CdTe) QDs.^95^ An aflatoxin B1 specific aptamer is conjugated to QDs and allowed to interact with AuNPs, quenching its fluorescence. The introduction of aflatoxin B1 removes the aptamer–QD conjugate from AuNPs and an increase in fluorescence is observed. However, the assay was able to detect aflatoxin at a higher concentration (10–400 nM) with a LoD of 3.4 nM, much higher than that in the previous original study, and the authors were able to sense the presence of aflatoxin in different food samples successfully.

### Raman based aptasensors

**Fig. 7:**
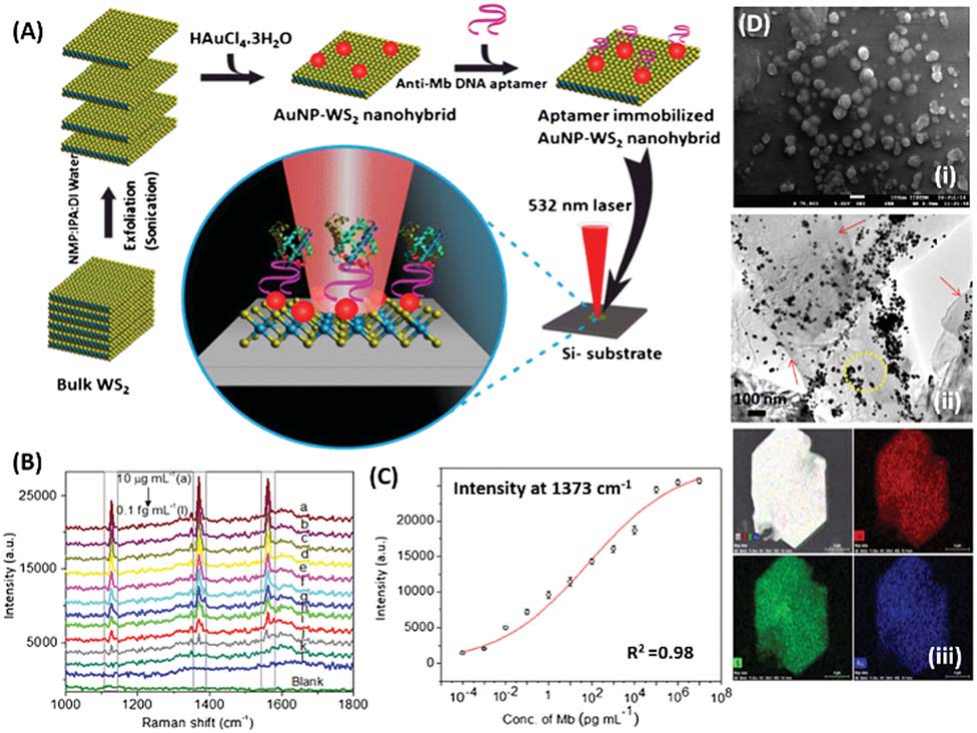
(A) Schematic representation of the synthesis of the WS_2_–AuNP SERS-active nanohybrid and its utilisation in the detection of myoglobin. (B) Raman spectra showing the increase in signal intensity in response to various concentration of myoglobin. (C) Fitting of signal intensity at 1373 cm^−1^. (D) Electron micrographs of the nanohybrid and its corresponding elemental mapping. Reprinted with permission from ref. 98. Copyright (2018) Springer Nature.

**Fig. 8:**
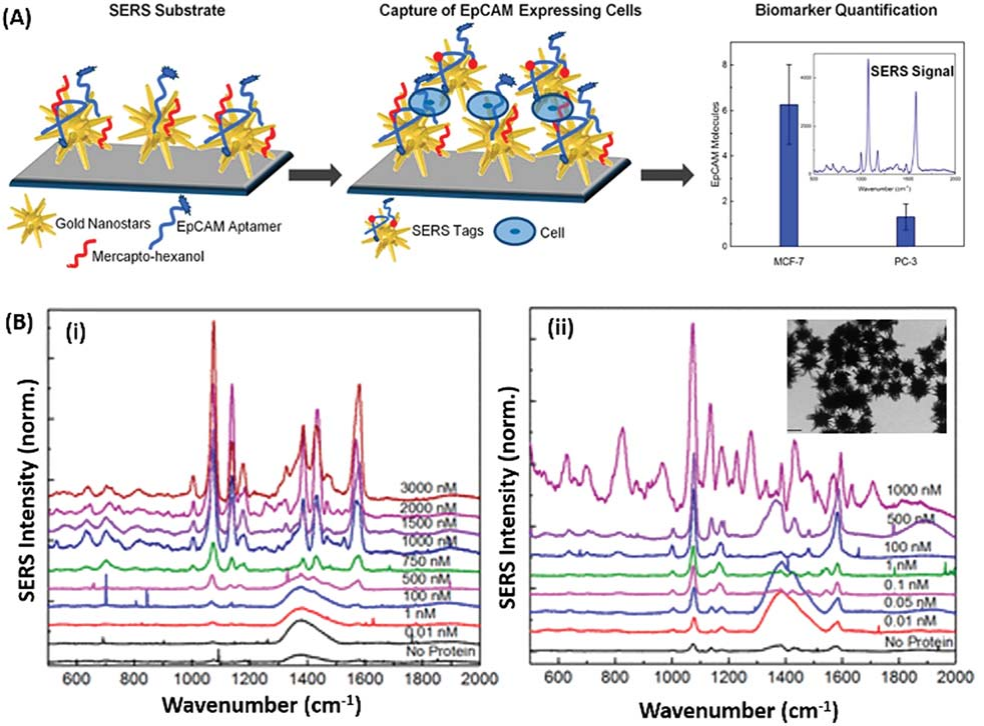
(A) Schematic representation of the gold nanostar based SERS platform for the detection of EpCAM. (B) SERS spectra collected on the substrate after exposure to increasing concentrations of EpCAM using (i) 48-mer and (ii) 17-mer EpCAM aptamers. Inset of (ii) shows transmission electron micrographs of gold nanostars after modification with the EpCAM aptamer. Adapted with permission from ref. 99. Copyright (2018) American Chemical Society.

Raman based aptasensors provide a more reliable sensing platform due to the measured signature signals of target moieties. Raman signatures are inelastic scattering of photons by molecules excited to higher vibrational or rotational energy levels. However, Raman signals are usually weak and are difficult to measure under normal conditions, thus limiting their application in assays. In recent times, the advancement in fabrication techniques have led to the development of Raman active “hot-spots” having significantly enhanced Raman signals. Such platforms usually utilize metallic nanostructures like gold or silver with inherent plasmons, which are enhanced multi-fold when the particles are in close proximity. This phenomenon called surface-enhanced Raman scattering (SERS) can be used as a label-free approach towards sensitive detection of compatible molecules. A silver nano-crown array based microfluidic system developed by Fu *et al.* is able to sense the Raman signal of 3,3′,4,4′-tetrachlorobiphenyl (PCB77) up to 10 nM which is captured by its specific aptamer bound on the silver nano-crown array.^96^ Dong *et al.* showed the ability of their SERS based aptasensor for the detection of melamine in milk samples utilized 4,4′ dipyridyl, a Raman tag bound to gold nanoparticles as their detection probe. The probe is lodged with tethering DNA, which binds to the platform modified with anchor DNA in a comparatively simpler format.^97^ Their developed platform detected the presence of melamine. The whole system utilizes the ability of melamine to crosslink thymine residues (T-melamine-T) among different DNA strands and allows sensing of the target molecule even in complex matrices like milk and milk powder. Furthermore, more recently Shorie *et al.* fabricated hotspots of gold nanoparticles on WS_2_ nanosheets by using the intrinsic reducing properties of the nanosheets (Fig. 7). A SERS active nanohybrid was modified with myoglobin specific aptamers and was able to detect the enhanced Raman signal from a cardiac protein in a range of 10 fg mL^−1^ to 0.1 μg mL^−1^.^98^ Recently, anisotropic metallic nanostructures are being employed in SERS based sensors due to their highly focused plasmonic hot-spots because of their morphologies. Bhamidipati & group showed the application of gold nanostars in such an assay for the detection of an epithelial cell adhesion molecule (EpCAM), a cancer biomarker (Fig. 8).^99^ Using the aptamer truncation method, they identified individual binding domains and further highlighted the role of different aptamer structures in developing detection platforms acting in different concentration ranges. The detection range of their developed SERS platform is between 10 pM and 500 nM, with potential application in single-cell detection.

### SPR based aptasensors

**Fig. 9:**
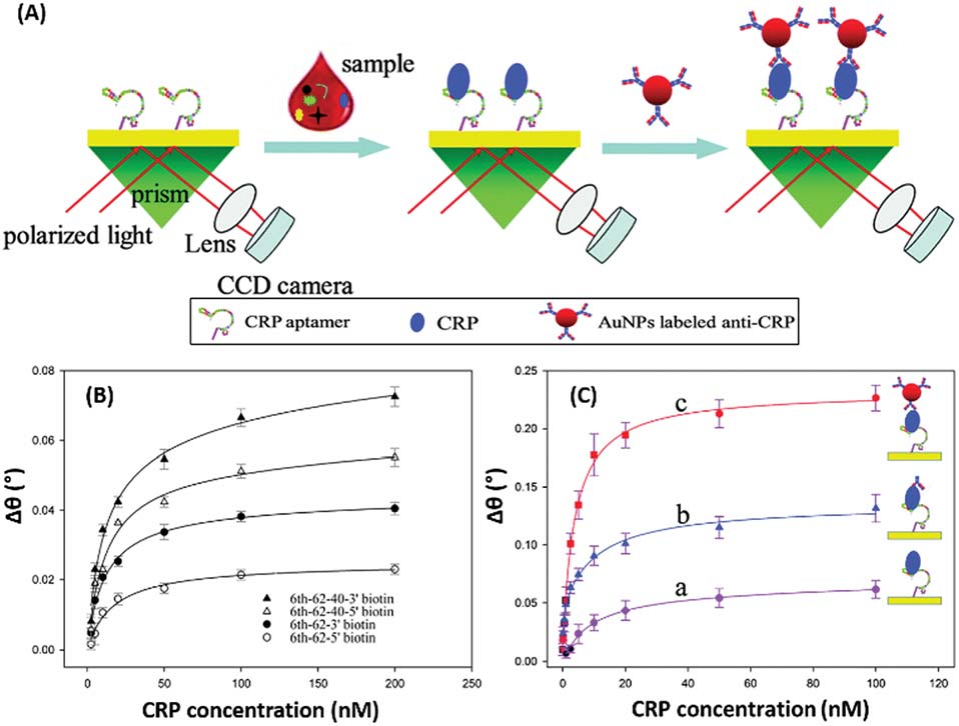
(A) Schematic illustration of an AuNP enhanced SPR biosensor for CRP with an aptamer–antibody sandwich assay. (B) The change in the resonance angle of immobilised aptamers at various concentrations of CRP. (C) Enhancement of the resonance angle by application of the antibody–AuNPs conjugate. Reproduced from ref. 100 with permission from the Royal Society of Chemistry.

**Fig. 10:**
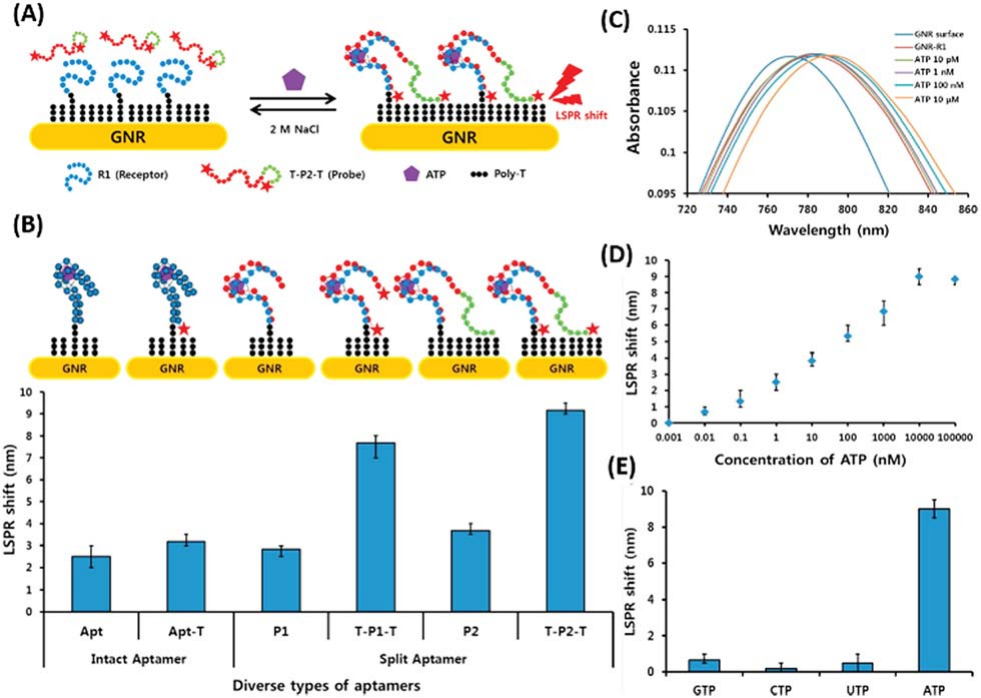
(A) Schematic representation of the developed LSPR aptasensor for ATP detection based on structure assembly inducing LSPR shift using split-aptamers labelled with TAMRA dye and a random sequence tail. (B) Optimisation of truncation of the aptamer and introduction of TAMRA into the aptamer to achieve maximum LSPR. (C) Shift in LSPR in response to various concentrations of ATP from 10 pM to 10 μM. (D) Linear fitting of the LSPR signals for ATP concentrations. (E) Specificity analysis of the developed LSPR based aptasensor for relevant nucleotides. Adapted with permission from ref. 103. Copyright (2015) Elsevier.

Surface plasmon resonance (SPR) is the collective vibrations of surface electrons of metallic structures. Being a surface phenomenon, SPR is very sensitive to any modifications occurring on the metal surface. This property has been utilized in the development of label-free SPR based aptasensors where the binding of analytes can be read as changes in the plasmon signals. However, SPR based sensors are limited by their evanescent fields and have a rather limited range in which they can detect molecules in conventional setups. However, Wu *et al.* developed a sandwich approach to overcome the inability of SPR sensors to detect smaller concentration as they are incapable of generating a readable shift in the plasmon (Fig. 9). In their developed sensor for C-reactive protein (CRP), they used aptamers as capture receptors to anchor CRP molecules on SPR chips and flowed anti-CRP antibodies bound to AuNPs. The sensor showed an ultrasensitive dynamic response between 10 pM and 100 nM in human serum samples.^100^ Furthermore Yoo *et al.* developed a localised surface plasmon resonance (LSPR) based chip from a glass slide by separating gold layers with a dielectric layer of silica nanoparticles for a multiplex detection assay for various bacterial species.^102^ For this, a glass slide was coated with a gold layer of 30 nm thickness *via* e-beam evaporation. The coated slide was further modified with 3 mm spots of activated silica nanoparticles before adding another 30 nm layer of gold. The separation of the gold layers creates a localisation of the surface plasmon, which further provides sensitivity to the platform as LSPR is more responsive to any changes occurring due to analyte binding in comparison to SPR, which is prominently affected by bulk changes.^101^ Aptamers specific to various bacteriological species were loaded on the chip *via* thiol–Au interactions and used to detect LSPR changes occurring due to binding of bacterial species on the chip *via* their aptamers. The chip showed a promising response in simultaneous detection of *E. coli*, *L. acidophilus*, *S. typhimurium* and *P. aeruginosa* up to 30 cfu per assay. Furthermore, for the development of LSPR sensors, Park *et al.* used gold nanorods as the LSPR source and carboxytetramethylrhodamine (TAMRA) as the detection probe.^103^ The binding pocket of an aptamer specific to adenosine triphosphate (ATP) was split into two fragments to fabricate an analyte-bridged receptor called a “split aptamer”, having one fragment docked on gold nanorods (GNRs) and the other bound to TAMRA (Fig. 10). With the use of split aptamers they were able to record LSPR shifts influenced by the presence of ATP caused by the changes in the distance between aptamer fragment-1 bound gold nanorods and aptamer fragment-2 bound TAMRA. The interaction affected the local microenvironment of the gold nanorods due to the changes in RI brought upon by TAMRA, which was further optimised by varying the length of TAMRA-bound fragment-2 to facilitate their interaction, thus increasing the sensitivity of the assay. The sensor showed a remarkable detection range of 10 pM to 10 μM and excellent reusability.

### Electrochemiluminescence based aptasensors

**Fig. 11:**
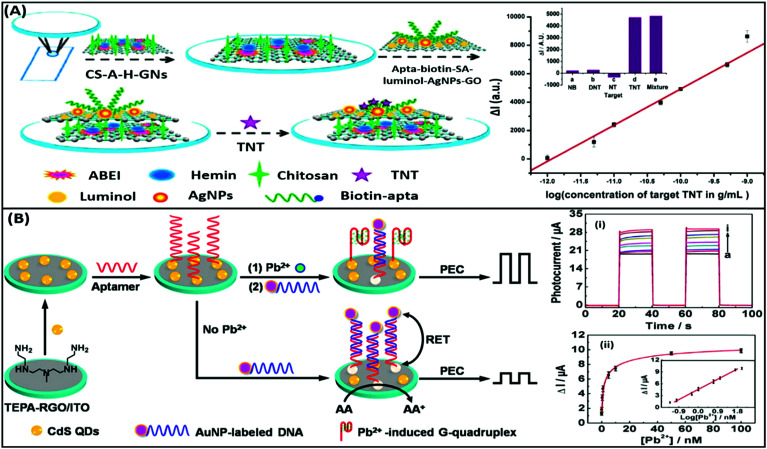
(A) Schematic illustration of the label-free ECL aptasensor for TNT detection based on an assembly strategy of ABEI–hemin–graphene hybrids (A–H–GNs) and luminol–AgNPs–GO. Plot shows the linear relative relationship between current and TNT concentrations. Inset shows the cross reactivity analysis of the developed system with nitrobenzene (NB), 2,4-dinitrotoluene (DNT) and *p*-nitrotoluene (NT). Adapted with permission from ref. 104. Copyright (2015) American Chemical Society. (B) Schematic diagram of stepwise modification of the ITO/RGO/CdS/aptamer sensing platform and “Signal-on” PEC detection strategy for lead ions on the basis of duplex–quadruplex conversion and RET between CdS QDs and AuNPs. Upper plot shows the increase in photocurrent upon the addition of lead ions. Legends a to i represent lead ion concentrations of 0, 0.05, 0.1, 0.5, 1, 5, 10, 50, and 100 nM. Lower plot shows the relationship between photocurrent and lead ion concentrations and its calibration plot (inset). Adapted with permission from ref. 107. Copyright (2014) American Chemical Society.

Electrochemiluminescence (ECL) is a type of luminescence in which the relaxation of an intermediate energy level excited by an electrical potential yields radiative energy. The generation of photons is correlated with the concentration of reacting analyte molecules present and thus presenting a more specific route of diagnosis. This method is applied in the development of aptasensors as the involvement of an electrochemical reaction creates a strict check on the signal generation giving a more selective and reliable response to the presence of analytes. ECL based detection platforms conventionally use a luminol–H_2_O_2_ system for the generation of electron–hole pairs, which lead to the generation of a radiant signal. An example of such detection platforms is depicted in the work of Li & group where they developed a complex bilayer aptasensing platform consisting of *N*-(aminobutyl)-*N*-(ethylisoluminol)/hemin dual-functionalized graphene hybrids (A–H–GNs) and a luminol functionalized silver/graphene oxide composite (luminol–AgNPs–GO).^104^ The platform works for the detection of 2,4,6-trinitrotoluene (TNT), which when present in the system quenches the luminescence by creating a pair with the aptamer, hindering the electron transfer (Fig. 11A). The decrease in the ECL signal enables the platform to detect TNT in a dynamic range of 1 × 10^−12^ to 1 × 10^−9^ g mL^−1^ with a limit of detection of 6.3 × 10^−13^ g mL^−1^. Alternatively, nanomaterials can be applied in ECL based detection platforms serving the purposes of energy transfer and conversion for signal generation. A study shown for detection of carbofuran utilizes the property of electrogenerated chemiluminescence resonance energy transfer (ECRET) between aptamer bound carbon dots and fullerene loaded gold nanoparticles.^105^ There is an efficient energy transfer between AuNPs (donor) and C-dots (acceptor) in a standard state ECL signal, which is compromised in the presence of carbofuran due to an increase in the distance between the donor and acceptor pair. The analyte-assisted conformational change in the carbofuran-specific aptamer creates a spatial gap insufficient for ECRET, which is used for the detection of the analyte in a concentration range of 2 × 10^−11^ to 8 × 10^−9^ mol L^−1^ with a LoD of 8.8 × 10^−13^ mol L^−1^. This technique can also be utilized for the detection of several other pesticide residue targets. Another study for the detection of lincomycin (a pesticide) uses the phenomenon of ECRET in a dual recognition system involving aptamers as well as molecularly imprinted polymers (MIPs).^106^ The system is built using a AuNP modified GO nanosheet composite, housing an aptamer/MIP dual recognition element bound to C-dots. Au–GO harvests and transfers the energy to C-dots acting as an acceptor and label moiety leading to a basal signal intensity of ECL. Lincomycin, when present in the solution, binds in the recognition pocket hindering the energy transfer which is manifested as a drop in the ECL signal corresponding to the pesticide concentration. A linear signal decline was obtained in a lincomycin concentration range of 5 × 10^−12^ to 1 × 10^−9^ mol L^−1^ with a LoD of 1.6 × 10^−13^ mol L^−1^ in buffers and meat samples. A unique property of ECL based systems over CL-based systems is their reverse application, which implies that electrical signal to light signal transduction can move in either direction. This property can be utilized for the generation of photoelectric current (PEC) where an acceptor molecule absorbs the radiant energy and generates an effective electron–hole pair. The electron is transferred to the detecting electrode and is measured as a change in the current signal. The phenomenon has gained attention in recent years over conventional ECL systems as a current signal has a lower probability of decay than luminescence increasing the credibility of the assay system. Zang & group showed the application of such PEC generation in a detection system for Pb^2+^ ions.^107^ The platform is fabricated from tetra-ethylene pentamine functionalised reduced graphene oxide nanosheets (TEPA–rGO), cadmium sulfide quantum dots (CdS QDs) and lead ion-specific aptamers added sequentially over an ITO electrode (Fig. 11B). The system is saturated with aptamer complementary DNA sequences bound to AuNPs, creating an efficient RET between AuNPs and CdS QDs. In the presence of Pb^2+^ ions the AuNP bound sequence is displaced from the system creating an upconversion of the PEC signal. The signal enhancement is linear between 0.1 and 50 nM ion concentration and showed good selectivity to Pb^2+^ ions in the presence of interfering ions and in environmental samples. Wang & co-workers showed a similar application of PEC generation in a graphene-like carbon nitride (gCN) based platform for the sensing of zeatin, a cytokinin.^108^ The system uses gCN as a photoactive material, graphene QDs as signal enhancers and AuNPs for immobilization of DNA aptamers. Zeatin-specific aptamers are covalently modified with biotin molecules and are bound to their complementary capture sequences in turn immobilized over a gCN/GQD/AuNP platform. Exonuclease I (a protein that cleaves ssDNA molecules) and streptavidin are sequentially introduced into the system. Streptavidin molecules act as PEC signal inhibitors and in the absence of zeatin hinder the generation of the PEC signal upon photo-irradiation. However, in the presence of zeatin, aptamers are displaced from their single stranded capture sequences eliminating the docking site of streptavidin and leading to an increase in the PEC signal in the process. Exonuclease I further enhances the PEC generation by cleaving and removing the single stranded capture molecules from the system. This intricate system is able to sense the presence of zeatin in a concentration range of 0.1–100 nM in a linear manner.

### Electrochemical aptasensors

Electrochemical sensors are a category of biosensors, which have an electrode acting as the transducing element. The changes occurring on the electrode surface because of biomolecular interaction can be measured to provide quantitative information. The current/voltage change in the redox reaction can be related to the concentration of the analyte or the rate of production/consumption over a specific time-period. Electrochemical sensors provide high sensitivity, robustness and ability to process complex matrices occurring in many biological samples. Developments in the field of microelectronics helped the expansion of the applicability of such sensors beyond the scope of laboratories by producing miniaturized handheld point-of-care (PoC) devices. Based on the measured parameter, electrochemical sensors can be broadly classified into **amperometric** and **impedimetric** sensors. Amperometric sensors measure changes in current as a measure of a variable parameter, usually time (chronoamperometry) or potential (voltammetry). Chronoamperometric sensors work by measuring current changes over time at a fixed potential in the redox reactions with respect to the analyte concentration.^109–111^ The technique is also compatible with non-electroactive analytes by incorporating enzymes, like oxidases, as in the most common glucose sensors, which can produce a readable current change by converting the analyte concentration into an electrical signal *via* electroactive mediators.^112–114^ Furthermore, such a method can also be used in a continuous measurement system by using enzymes as receptor molecules as well as transducers as they are not depleted during the interaction and signal generation.^115^ However, due to its limited applicability, chronoamperometry is less used among electrochemical techniques and thus is not discussed in detail in the further text. Voltammetric techniques on the other hand are more versatile due to the involvement of variable system potential and have been applied in the development of a large number of distinct diagnostic approaches. Impedimetric sensors in contrast rely on the changes occurring in the charge transfer abilities of the interface as a result of the biointeractions, and have grabbed the attention of the research community due to their label-free nature when used in combination with aptamers.

The discovery of graphene marked the beginning of an era of advanced nanomaterials with very high electronic properties.^116^ Further research in the synthesis of materials helped the expansion of the electroactive nanomaterial class by adding several members like planar transition metal chalcogenides (TMCs), metal oxides, carbides and nitrides, and more recently pnictogens and topological insulators. Electroactive materials are used as the electrode material in such assays and have helped push the sensitivities to very low analyte concentrations due to their superior electrochemical properties. Examples of such nanomaterial based assays are listed in Table 1.

**Table 1:** List of various nanomaterials applied as transduces for the development of aptasensors for various clinical and environmental analytes

Transducer	Nanomaterial	Analyte	Range of detection	Limit of detection	Ref.
Colorimetric	AuNPs	Ochratoxin A	50–1000 nM	1.27 nM	54
	AuNPs	17β-estradiol	0.2 nM to 0.2 mM (17β-ED)	0.2 nM (17β-ED)	55
		Cocaine	1–100 nM (Coc)	1 nM (Coc)	
		Ochratoxin A	1 nM to 10 μM (OTA)	1 nM (OTA)	
	AuNPs	ATP	50–1000 nM	50 nM	56
	AuNPs	VEGF	185 pM to 7.4 nM	185 pM	57
	AuNPs	Microcystin-LR	0.5 nM to 7.5 μM	0.37 nM	58
	AuNPs	17β-estradiol	200–800 pM	200 pM	59
	AuNPs	Acetamiprid	0.3–5 μM	0.4 μM	60
	AuNPs	Acetamiprid	0.1–100 ppm	0.1 ppm	61
	AgNPs	As^3+^ ions	50–700 μg L^−1^	6 μg L^−1^	62
	AgNPs@GO	Hg^2+^ ions	10–200 μM	338 nM	63
	AgNPs	Kanamycin	0.05–0.6 μg mL^−1^	2.6 ng mL^−1^	64
Fluorescence	GO	Bisphenol A	0.1–10 ng mL^−1^	0.05 ng mL^−1^	73
	GO	Thrombin	5–1200 nM	1.8 nM	74
	GO	Theophylline	1–100 μM	0.155 μM	75
	MoS_2_	Prostate specific antigen	0.5–300 ng mL^−1^	0.2 ng mL^−1^	78
	AuNPs	Theophylline	0.1–10 μM	0.05 μM	79
	AuNPs	Neomycin B	0.1–10 μM	0.01 μM	80
	Mo_2_C nanotubes	Bisphenol A	2–20 nM	2 nM	81
	CQDs	Myoglobin	1 to 10^5^ ng mL^−1^	1 ng mL^−1^	89
	CQDs	*S. typhimurium*	10^3^ to 10^5^ cfu mL^−1^	50 cfu mL^−1^	90
	AgNC	Pb^2+^ ions	5–50 nM	3 nM	92
	GQDs/GO	Pb^2+^ ions	9.9–435 nM	0.6 nM	93
	C-dots/AuNPs	Aflatoxin B1	16 pM to 6.4 nM	16 pM	94
	CdTe QDs/AuNPs	Aflatoxin B1	10–400 nM	3.4 nM	95
Raman	Silver nanocrowns	PCB77	1 × 10^−4^ to 1 × 10^−8^ M	1 × 10^−8^ M	96
	AuNPs	Melamine	1 pg mL^−1^ to 10 ng mL^−1^	1 pg mL^−1^	97
	Au@WS_2_	Myoglobin	10 fg mL^−1^ to 0.1 μg mL^−1^	10 fg mL^−1^	98
	Au nanostars	EpCAM	10 pM to 500 nM	10 pM	99
SPR	AuNPs	CRP	10 pM to 100 nM	10 pM	100
	Au@Si	Bacteria	10^4^ to 10^9^ cfu mL^−1^	10^4^ cfu mL^−1^	102
	Au nanorods	ATP	10 pM to 10 μM	10 pM	103
ECL	Graphene hybrids	TNT	1 × 10^−12^ to 1 × 10^−9^ g mL^−1^	6.3 × 10^−13^ g mL^−1^	104
	C-dots/AuNPs	Carbofuran	2 × 10^−11^ to 8 × 10^−9^ mol L^−1^	8.8 × 10^−13^ mol L^−1^	105
	AuNPs–GO/C-dots	Lincomycin	5 × 10^−12^ to 1 × 10^−9^ mol L^−1^	1.6 × 10^−13^ mol L^−1^	106
	AuNPs–rGO/CdS QDs	Pb^2+^ ions	0.1–50 nM	0.05 nM	107
	gCN/GQD/AuNPs	Zeatin	0.1–100 nM	0.031 nM	108
CV	rGO/CNT	Myoglobin	1 ng mL^−1^ to 4 μg mL^−1^	0.34 ng mL^−1^	117
	Phosphorene	Myoglobin	1 pg mL^−1^ to 16 μg mL^−1^	0.524 pg mL^−1^	118
	SWCNTs	Cocaine	0.1–50 nM	136 pM	119
DPV	MoSe_2_–graphene	PDGF	0.1 pM to 1 nM	0.02 pM	120
	AuNPs	β-Amyloids	0.5–30 nM	0.1 nM	122
	rGO/PEI	BNP/TnI	1 pg mL^−1^ to 1 μg mL^−1^ (TnI)	1 pg mL^−1^	123
			1 pg mL^−1^ to 10 ng mL^−1^ (BNP)		
	rGO	Aflatoxin B1	0.4 nM to 4 μM	0.07 nM	124
SWV	Graphene	β-Lectoglobulin	100 pg mL^−1^ to 100 ng mL^−1^	20 pg mL^−1^	125
	MnO_2_ nanosheets	Cocaine	0.1–20 nM	32 pM	126
	Metal–organic framework	Kanamycin, chloramphenicol	0.002–100 nM	0.16 pM (Kan)	127
				0.19 pM (Chlor)	
EIS	AuNPs	Interleukin (IL-6)	0.02–20 pg mL^−1^	0.02 pg mL^−1^	128
	AuNPs/MWCNT/graphene nanoribbons	Acetamiprid	5 × 10 ^−14^ to 1 × 10^−5^ M	1.7 × 10^−14^ M	129
	Ordered mesoporous carbon–Au nanocomposite	Vascular endothelial growth factor-165	10–300 pg mL^−1^	1 pg mL^−1^	130
	Graphene	Thrombin	0.3–50 nM	0.01 nM	131
	AuNPs	MUC-1	0.1–10 nM	0.1 nM	132
	Bridged rebar graphene	*E. coli*	10^1^ to 10^6^ cfu mL^−1^	10^1^ cfu mL^−1^	133
	AuNPs	*S. dysenteriae*	10^1^ to 10^6^ cfu mL^−1^	10^0^ cfu mL^−1^	134
Mechanical	AuNPs	Pb^2+^ ions	5–200 nmol L^−1^	4 nmol L^−1^	136
	AuNPs	Thrombin	0.1–10 μM	0.1 μM	137
	AuNPs	Thrombin	10^−18^ to 10^−13^ M	0.78 aM	138
	Graphene	Thrombin	5–1000 ng mL^−1^	5.57 ng mL^−1^	139
	Graphene	*S. aureus*	4.1 × 10^1^ to 4.1 × 10^5^ cfu mL^−1^	41 cfu mL^−1^	140

### Voltammetric aptasensors

**Fig. 12:**
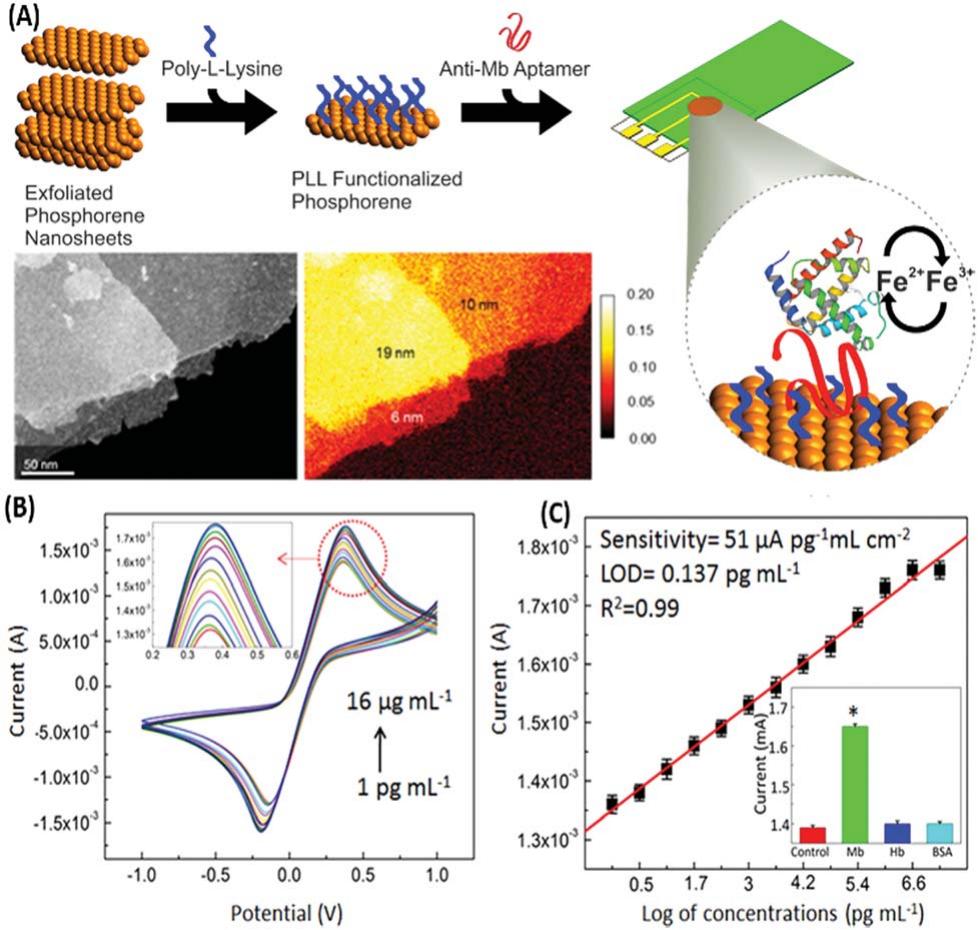
(A) Schematic representation of the development of a phosphorene based electrochemical platform for myoglobin. Inset shows the transmission electron micrograph and elemental mapping of the exfoliated nanosheets. (B) Increase in the current response in a concentration-dependent manner for various concentrations of myoglobin as measured with cyclic voltammetry. (C) Linear fitting of the oxidation peak current in response to myoglobin concentrations. Inset shows cross-reactivity analysis with serum proteins. Adapted with permission from ref. 118. Copyright (2015) American Chemical Society. Further permissions related to material excerpted should be directed to the ACS.

Voltammetric sensors measure the variations in the current signal over a variable potential range as an indication of analyte concentration. Different variations of voltammetry *viz.* cyclic voltammetry (CV), differential pulse voltammetry (DPV) and square wave voltammetry (SWV) have been applied for the detection of various types of molecules in combination with the selectivity provided by aptamers. In a simple label-free assay format Kumar & co-workers showed the detection of an electroactive protein and cardiac biomarker, myoglobin.^117^ The assay utilized the inherent redox switch occurring in the myoglobin on a rGO/carbon nanotube nanohybrid modified screen printed electrode (SPE) *via* CV. Another study on direct electron transfer based assay for myoglobin is shown on a few-layer phosphorene modified electrode using CV with a very wide range of detection (1 pg mL^−1^ to 16 μg mL^−1^) (Fig. 12).^118^ The detection of non-electroactive molecules requires the involvement of a conducting label like ferrocene or methylene blue which give current response respective to their specific potential. Single walled carbon nanotubes (SWCNTs) are found to be a promising candidate for such assays as shown by Taghdisi & group for the detection of cocaine.^119^ In their assay, a complementary DNA strand is bound on a gold electrode which can host anti-cocaine aptamers. The introduction of cocaine displaces the aptamer, leaving the complementary strand available for binding to SWCNTs, which increases the electrochemical response in CV relative to the cocaine concentration.

**Fig. 13:**
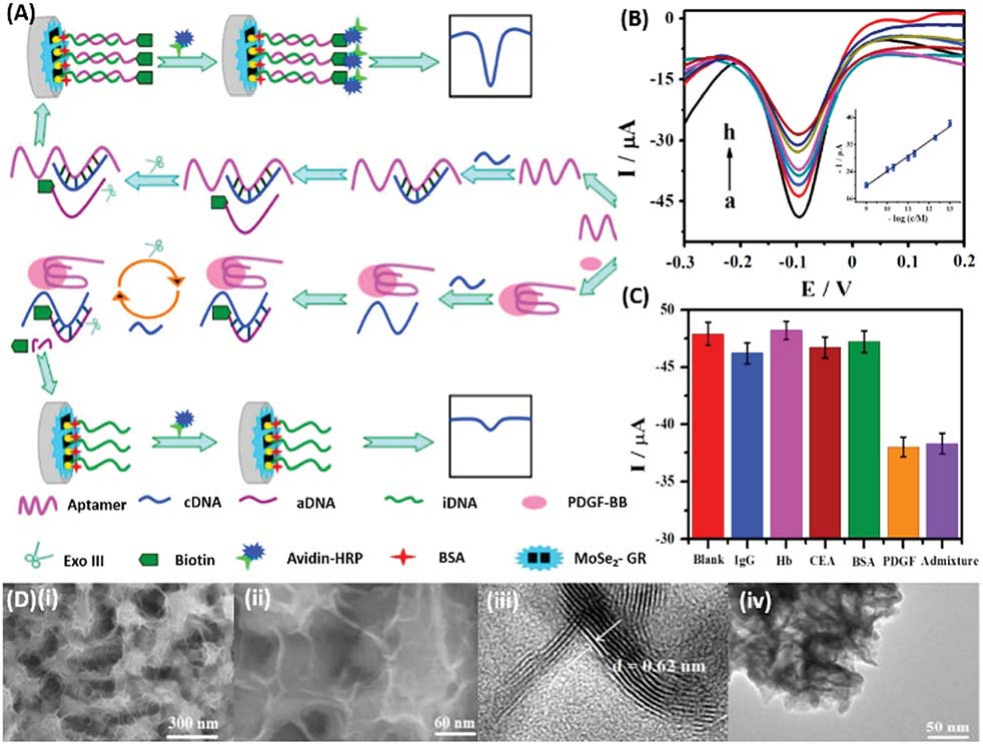
(A) Schematic illustration of biosensor construction and its application for PGDF-BB detection with exonuclease III assisted signal amplification. (B) DPV curves of various PGDF-BB concentrations ranging from 1 × 10^−13^ to 1 × 10^−9^ mol L^−1^ (a–h). Inset shows the calibration plot for DPV currents *vs.* log concentration of PGDF-BB. (C) Amperometric response of the developed aptasensor in the presence of related serum proteins *viz.* immunoglobin G (IgG), haemoglobin (Hb), carcinoembryonic antigen (CEA), and bovine serum albumin (BSA). (D) Transmission electron micrographs showing the MoSe_2_–graphene nanohybrid. Adapted with permission from ref. 120. Copyright (2016) Elsevier.

**Fig. 14:**
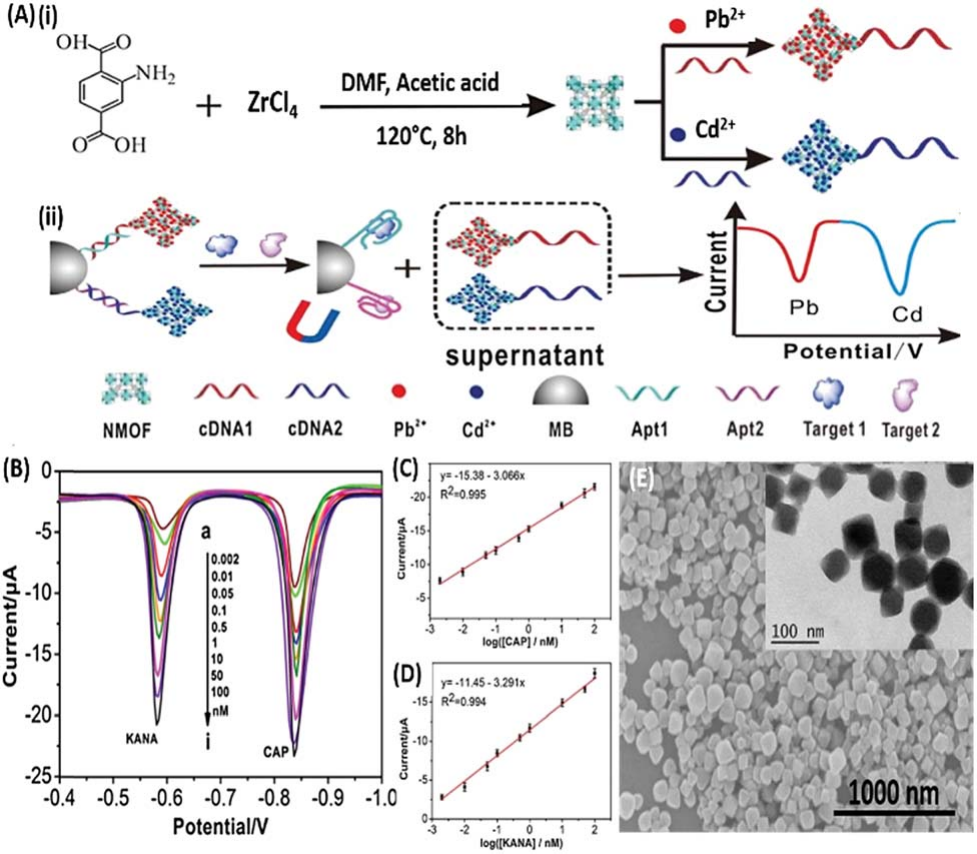
(A) Schematic illustration of (i) synthesis of UiO-66-NH_2_ nanoparticles and preparation of signal probes, and (ii) their usage in simultaneous electrochemical detection of kanamycin and chloramphenicol based on aptamer-nanoscale MOFs as biocodes. (B) Dual SWV signals obtained from the developed platform in the presence of various concentrations of antibiotics from 0.002 (a) to 100 nM (i). (C) Calibration curve for kanamycin showing a linear signal increase. (D) Calibration curve for chloramphenicol showing a linear signal increase. (E) Scanning electron micrograph of the synthesized MOF nanoparticles showing the surface topology. Inset shows individual MOF particles as visualised under TEM. Adapted with permission from ref. 127. Copyright (2017) Elsevier.

DPV is the more frequently used technique in such assays as demonstrated by Huang's group & Hamidi-Asl's group in the detection of the platelet derived growth factor (PDGF) and chloramphenicol respectively (Fig. 13).^120,121^ The use of dual receptors in a DPV format is demonstrated by Zhou & colleagues for the detection of beta-amyloid oligomers.^122^ The assay uses an amyloid specific antibody and an aptamer in a sandwich manner and the signal of thionine conjugated to the aptamer is measured at −0.1 V potential by DPV. In another study, Grabowska & colleagues demonstrated the application of a single detection platform fabricated from a nanohybrid of rGO and polyethylenimine (rGO/PEI) in the detection of structurally different cardiac biomarkers *viz.* B-Type natriuretic peptide (BNP) and troponin I (TnI) *via* DPV. The assay for the first time established the fact that a single platform can be used for multiple markers by switching between specific aptamers.^123^ In a more recent study, unique application of rGO as a signal amplifier was demonstrated by Beheshti-Marnani & research group.^124^ The detection platform uses a rGO–aptamer conjugate as the label element, which is able to detect Aflatoxin B1 in a low concentration range of 0.4 nM to 4 μM with a detection limit of 0.07 nM. Similar to other voltammetric techniques, SWV also provides the advantage of signal specificity as different labels generate signature signals at unique potential values, thus creating a dual recognition mode alongside aptamers. As shown in the work of Eissa *et al.* SWV is exploited in the detection of β-lectoglobulin, an allergen found in complex matrices of milk samples.^125^ Their aptasensor is able to detect the presence of the analyte in a linear concentration range of 100 pg mL^−1^ to 100 ng mL^−1^ with minimal cross reactivity among interfering species. A similar application is shown in the work of Chen and Lu on MnO_2_ nanosheets using ferrocene as an electrochemical label.^126^ The signal of ferrocene conjugated to an anti-cocaine aptamer over MnO_2_ nanosheets is recorded, which depletes in the presence of cocaine due to strand displacement. The decline is linear between 0.1 and 20 nM and the limit of detection was found to be 32 pM. The property of signature SWV signals allows their utilisation in multiplex systems where the usage of separate labels generates distinct signals. The excellent application of SWV signatures was shown in the work of Chen & co-workers for dual detection of kanamycin and chloramphenicol simultaneously.^127^ In a SWV setup the platform uses lead and cadmium metal ions embedded in nanoscale molecular organic frameworks (MOFs). These metal–MOF conjugates are linked to antibiotic specific aptamers creating label elements and the signature signal of each metal ion is measured as an indication of antibiotic concentration (Fig. 14). The assay system is able to detect the presence of both antibiotics between 0.002 and 100 nM with a LoD of 0.16 pM for kanamycin and 0.19 pM for chloramphenicol.

### EIS based aptasensors

**Fig. 15:**
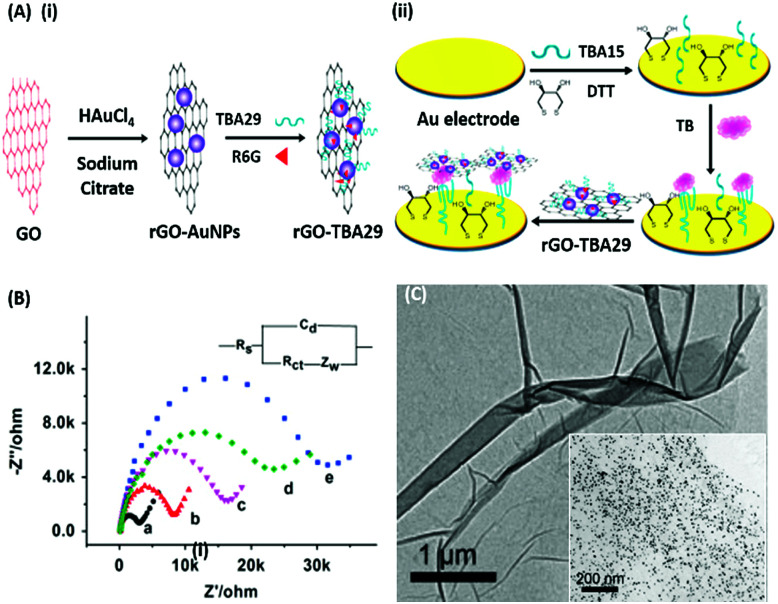
(A) Schematic illustration of (i) synthesis of rGO–AuNPs–aptamer conjugates and (ii) development of a signal amplified impedimetric aptasensing platform for thrombin (TB) detection. (B) EIS responses of the developed platform for various concentrations of thrombin. Inset shows the Randles–Sevcik circuit diagram applied to obtain Nyquist plots. (C) Transmission electron micrographs showing rGO and the rGO–AuNP conjugate (inset). Adapted with permission from ref. 131. Copyright (2015) Elsevier.

**Fig. 16:**
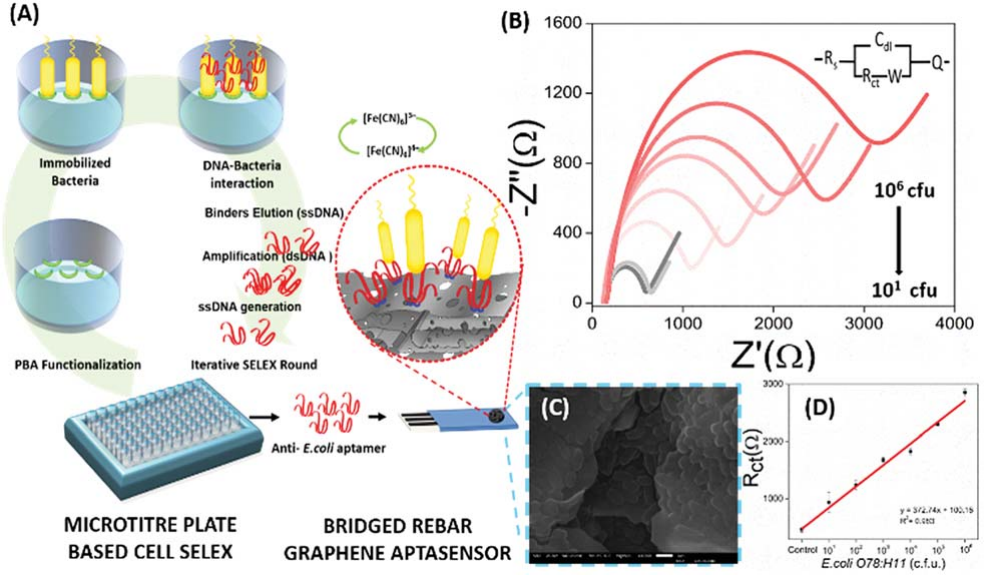
(A) Schematic representation showing the steps of microtitre plate based cell-SELEX and functionalization of the bridged rebar graphene (BRG) coated screen-printed electrode (SPE) with a screened anti-*E. coli* aptamer. (B) Nyquist plot showing the increase in *R*_ct_ with the inclusion of *E. coli O78:H11* cells. The plot has been fitted using the Randles–Sevcik equation (inset). (C) SEM image showing the surface of the aptasensor with captured bacteria and (D) a calibration curve for the aptasensor showing a linear increase in sensor measurements. Adapted with permission from ref. 133. Copyright (2017) Elsevier.

Electrochemical Impedance spectroscopy (EIS) has attracted the attention of researchers due to its ease of designing, quick assay format, simplicity and cost effectiveness. Aptamers have been integrated into impedance-based assays providing essential selectivity while keeping the basal resistance low enough for analyte measurement, an important prerequisite severely lacking in antibodies. In contrast to other methods, EIS measures the conductivity of the system over a wide frequency sweep range of 10 kHz to 10 MHz as a measure of the substrate concentration. The main advantage of EIS is its label-free nature, which increases its applicability in the detection of non-electroactive substrates, providing EIS an edge over other alternative electrochemical methods.^128–130^ The work of Wang *et al.* is rather novel for the use of a nanomaterial as the label in an EIS sensor.^131^ A thrombin specific aptamer (TBA15) covalently bound to the electrode surface captures the analyte molecule while another anti-thrombin aptamer (TBA29) conjugated to rGO and rhodamine 6G (R6G) is used as a label binding in a sandwich manner, thus hindering the charge transfer (Fig. 15). Gold nanoparticles have been employed in the same manner by Liu & co-workers for the detection of Mucin-1 (MUC-1), a cancer biomarker.^132^ The platform uses an AuNP modified aptamer as a label element which in the presence of MUC-1 between 0.1 and 10 nM increases the impedance of the system linearly. The technique can be successfully applied for several other molecules possessing inferior ability of creating insulating layers themselves in EIS setups. The decrease in the conductivity of the electroactive surface depends on the efficacy of the analyte in creating an insulating layer, thus making the EIS based sensors superior in the detection of large targets like whole cells (Fig. 16). The application of EIS in the ultrasensitive detection of bacterial pathogens *E. coli* and *S. dysenteriae* has been a proof in such cases.^133,134^

### Mechanical aptasensors

**Fig. 17:**
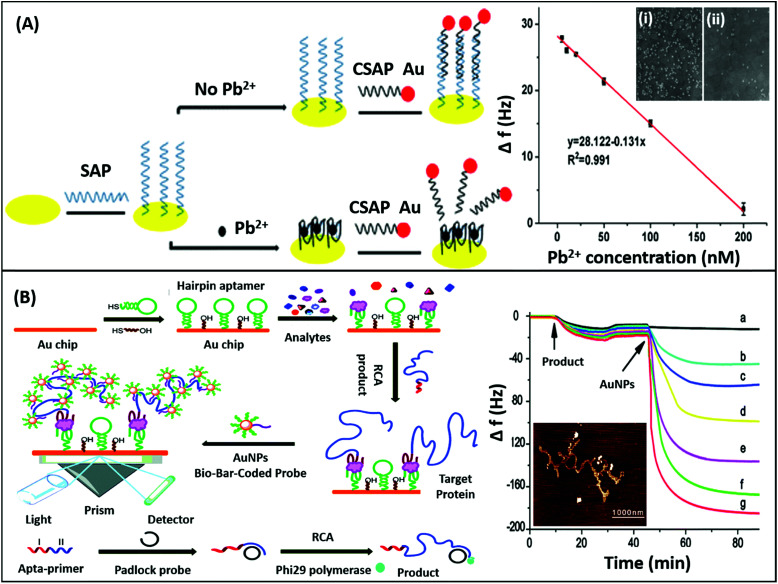
(A) Schematic showing the fabrication of a QCM aptasensor for Pb^2+^ detection based on the usage of specific aptamers immobilized onto the surface of quartz crystals and the binding of Pb^2+^, which prevents the self-assembly of AuNPs on the microbalance. Here, SAP denotes specific aptamer and CSAP denotes partially complementary aptamer. The sensor shows a linear relationship between frequency drop and target concentration. Inset shows the SEM images of the crystal in the absence (i) and presence (ii) of lead ions. Reprinted with permission from ref. 136. Copyright (2017) Springer Nature. (B) Schematic representation of the SPR assay for α-thrombin detection using a sandwich aptamer recognition mechanism and cascade signal amplification by aptamer-based RCA and bio-bar-coded AuNP enhancement, which was confirmed by the real-time frequency responses of the amplified QCM biosensor in the presence of thrombin. From (a) to (g): 10^−18^, 10^−17^, 10^−16^, 10^−15^, 10^−14^, and 10^−13^ M thrombin. The AFM image of the RCA product obtained *via* amplification is shown in the inset. Reproduced from ref. 138 with permission from the Royal Society of Chemistry.

Mechanical sensors are the most advanced category of biosensors with the highest sensitivities and are rapidly gaining interest with increasing research on the physical nature and mechanical properties of biomolecules.^135^ The mechano-transductory interactions occurring on the sensor surface are monitored *via* changes occurring in the physical properties of the sensors, usually cantilevers, piezoelectric materials or quartz crystal microbalances (QCMs). The interaction of the analyte with the sensor-bound receptor affects the mass of the system which is transduced differently by different sensors. The biological interaction causes deflection of the signal due to bending of the sensor in cantilevers, whereas it changes the conducting properties of the piezoelectric sensor due to the strain caused by the gain in mass, and affects the oscillations of the crystal in the QCM. Although the application of nanomaterials has been difficult in such sensors conventionally as to avoid gain of background mass, several unique mechanical sensors have been fabricated in recent years displaying innovative use of nanomaterials for the biosensing of analytes. QCMs represent the most popular category extensively utilising nanomaterials, usually gold nanoparticles. As shown in the work of Yuan & co-workers for the detection of lead ions, AuNPs are used in a non-orthodox manner (Fig. 17A). Lead ions, which are not capable of generating enough signal change due to small mass, are tested by using an aptamer modified QCM crystal. The aptamer-QCM is saturated with a complementary DNA–AuNP conjugate, which in the presence of lead ions is displaced, thus creating a net loss in mass.^136^ The technique is novel and can be efficiently employed for several other low mass moieties. Furthermore, Deng and co-authors developed a method for dual mode detection of thrombin using AuNPs. It was proposed that AuNPs increase the sensitivity of QCMs by offering a larger surface area for aptamer modification and simultaneously provide a route for SERS based detection of thrombin. The assay further assessed the effect of an AuNPs–aptamer conjugate based label complex on the sensitivity of the assay and found that such a label is able to cause a 10-fold improvement in the detection limits of the assay.^137^ A similar technique of AuNP based signal enhancement was employed by He and colleagues in an assay for the detection of thrombin, where an AuNP based label was used to increase the mass gain and thus increase the signal intensity and assay sensitivity.^138^ Being a mass-based technique, the authors performed a simultaneous detection *via* SPR in another dual-mode aptasensor increasing the reliability of the sensor (Fig. 17B). Besides AuNPs, graphene has been employed in QCM based sensors due to its mechanical properties besides the high surface area it offers. In an article by Zhang & group, a graphene and plasma polymerized allylamine (PPAA) composite was used as the detection platform for label-free quantification of thrombin simultaneously by using QCMs and EIS.^139^ They experimentally reported an enhancement in the mechanical properties of graphene by using polymerised allylamine and were able to monitor the binding kinetics of thrombin on a graphene–PPAA nanofilm suggesting its possible usage in gene-therapy and protein detection. Another application of graphene in mechanical sensors was in the development of piezoelectric aptasensors for the detection of *Staphylococcus aureus*.^140^ In a displacement setup, aptamers specific to *S. aureus* bound to the graphene-modified sensor are removed in the presence of bacteria causing a decrease in mass and changing the properties of graphene in the process. The label-free assay has a detection range of 4.1 × 10^1^ to 4.1 × 10^5^ cfu mL^−1^ with a LoD of 41 cfu mL^−1^ and can detect target bacteria in clinical and food samples in a rapid and specific manner. Although mechanical biosensors have higher developmental cost, recent improvements in the field of mechanical PoC devices have paved the way for the applicability of such biosensors.

## Summary and future outlook

The review encompasses a wide array of nanomaterials ranging from the versatile 0D quantum dots, metallic nanostructures like crowns and ribbons to 2D nanomaterials like phosphorene, WS_2_ and a multitude of nanocomposites and 3D nanostructures. Recent research highlighting these novel nanomaterials in conjugation with aptamers specific to clinical and environmental targets has opened avenues in the healthcare and food industries. As an advancement of conventional diagnostics, these aptamer-modified nanostructures have been extensively worked upon and perhaps will displace other detection platforms in a short time. This review categorized aptasensing nanoplatforms based upon all available detection techniques developed by various researchers in recent five years, giving a broader understanding about advances in this fast-paced diagnostics market. This also brings to view that exploitation of different properties of the same nanomaterial like gold nanoparticles, graphene and its hybrids can be utilized for the fabrication of transducers towards multiple targets with the help of aptamers. High affinity aptamers can be generated for analytes ranging from small molecules to whole cells and a deeper insight for their application in diagnostic approaches is necessary. Furthermore, better integration of nanomaterials and aptamers for multiplex detection of analytes in a facile and cost-effective setup especially for PoC diagnosis needs to be explored in depth. The current need of the hour is sensitive detection coupled with easy to handle devices that may aid in monitoring of clinical samples like blood, urine and environmental and food samples with ease and future progress in integrated PoC devices is awaited.

## Conflicts of interest

There are no conflicts to declare.

## Supplementary Material
